# Epidemiology and aetiology of dialysis-treated end-stage kidney disease in Libya

**DOI:** 10.1186/1471-2369-13-33

**Published:** 2012-06-08

**Authors:** Wiam A Alashek, Christopher W McIntyre, Maarten W Taal

**Affiliations:** 1 School of Graduate Entry Medicine, University of Nottingham, Nottingham, UK; 2 Department of Renal Medicine, Derby Hospitals NHS Foundation Trust, Derby, UK; 3 Royal Derby Hospital, Uttoxeter Road, Derby, DE22 3NE, UK

**Keywords:** Dialysis, Epidemiology, ESKD, Incidence, Libya, Prevalence

## Abstract

**Background:**

The extent and the distribution of end stage kidney disease (ESKD) in Libya have not been reported despite provision of dialysis over 4 decades. This study aimed to develop the first comprehensive description of the epidemiology of dialysis-treated ESKD in Libya.

**Methods:**

Structured demographic and clinical data were obtained regarding all adult patients treated at all maintenance dialysis facilities (n=39) in Libya from May to September 2009. Subsequently data were collected prospectively on all new patients who started dialysis from September 2009 to August 2010. Population estimates were obtained from the Libyan national statistics department. The age and gender breakdown of the population in each region was obtained from mid-2009 population estimates based on 2006 census data.

**Results:**

The prevalence of dialysis-treated ESKD was 624 per million population (pmp). 85% of prevalent patients were aged <65 years and 58% were male. The prevalence of ESKD varied considerably with age with a peak at 55–64 years (2475 pmp for males; 2197 pmp for females). The annual incidence rate was 282 pmp with some regional variation and a substantially higher rate in the South (617 pmp). The most common cause of ESKD among prevalent and incident patients was diabetes. Other important causes were glomerulonephritis, hypertensive nephropathy and congenital or hereditary diseases.

**Conclusions:**

Libya has a relatively high prevalence and incidence of dialysis-treated ESKD. As the country prepares to redevelop its healthcare system it is hoped that these data will guide strategies for the prevention of CKD and planning for the provision of renal replacement therapy.

## Background

End-stage kidney disease (ESKD) is highly prevalent globally. It has become a major public health problem and is associated with considerable co-morbidity and mortality. Maintenance dialysis therapy is the commonest mode of renal replacement therapy and demand for this service is increasing progressively worldwide.

Libya is a sparsely populated medium-developed country but it has a high prevalence of risk factors for chronic kidney disease (CKD) such as diabetes, hypertension and obesity [1-4]. Societal, economic and environmental transformation have contributed to people tending to adopt a sedentary life [5]. Attention paid by the primary health care systems to combat the rising epidemic of chronic diseases has been inadequate [6]. In contrast, Libya was among the first countries in the region to establish free access to maintenance dialysis therapy for patients with ESKD [7]. Health care administrative bodies have continued to expand dialysis services in terms of geographic coverage and capacity to cope with increasing demand [7]. Kidney transplantation in Libya is limited by the lack of cadaveric donors and limited availability of suitable living-related donors [8,9]. Thus the majority of patients with ESKD remain dialysis dependent. Nevertheless, data regarding the epidemiology of ESKD and dialysis treatment in Libya are scarce and knowledge about the spectrum of renal diseases is very limited. The purpose of this study was to develop the first comprehensive description of the epidemiology of dialysis-treated ESKD in Libya. The study was performed prior to the recent conflict but as Libya prepares to redevelop its healthcare system these data will be vital to guide strategies for the prevention of CKD and planning for the provision of renal replacement therapy.

## Results

### Prevalence of ESKD in Libya

As shown in Table 1, the total number of adult ESKD patients undergoing maintenance dialysis therapy in Libya was 2417 in August 2009. The estimated adult population of Libya during 2009 was 3,873,000, giving a prevalence of dialysis-treated ESKD of approximately 624 per million population (pmp). The prevalence rate varied slightly by region with the highest rate of 628 pmp in North West region, the most populated area of the country. Most prevalent patients were under 65 years of age (85%). Female patients tended to be older than males, except in the South. Duration of dialysis was a median of 3 years and tended to be lower in females (Table 2). The majority of dialysis patients were Libyan nationals (97.8% of prevalent and 96.6% of incident patients).

**Table 1:** Prevalent and Incident dialysis patient numbers and rates for Libya and its regions

	**Regions**			**Whole country**
	**North West**	**North East**	**South**	
Number of prevalent patients 2009	1558	653	206	2417
Prevalence rate 2009 (pmp)	628	623	597	624
Number of incident patients 2010	593	287	213	1093
Incidence rate 2010 (pmp)	239	274	617	282

**Table 2:** Age, age at onset and dialysis vintage for prevalent and incident dialysis patients in Libya and its regions. Data are median and interquartile range

		**Region**			**Total/gender**	**Total**
		**North West (n=1558)**	**North East (n=653)**	**South (n=206)**		
Age of prevalent	Male	49	46	49	48	
patients in years		36-61	35-59	36-61.3	36-61	49
(n=2417)	Female	49	50.5	42.5	49	36-61
		34-60	37-61	32.3-59	35-61	
Age at onset of dialysis	Male	45	42	47	44	
(n=2417)		31-58	28-55	31-59.9	30-58	45
	Female	45	46	38	45	30-58
		30-57	31-57	27-57.8	30-57	
Dialysis vintage in years	Male	3	3	2	2.5	
(n=2417)		1-6	1-7	0.1-4	1-6	3
	Female	3	3	2	3	1-6
		1-5	1-6	2-2	1-6	
Age of incident patients	Male	50	47	46	48	
in years (n=1093)		37-63	34-61	30-57	35-61	49
	Female	52	53	43	50	35-62
		35-66	41-64	36-54	36-64	

**Figure 1:**
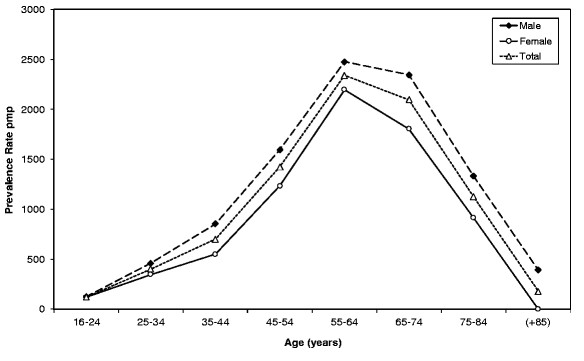
Prevalence rate pmp of dialysis-treated ESKD in Libya for males and females by age group.

Figure 1, shows that the prevalence of dialysis-treated ESKD was higher among males versus females at all ages. Overall, males represented 58% of prevalent dialysis population. The prevalence of ESKD varied considerably with age. Prevalence rates were low in young adults but showed a steady increase with age. Prevalence rates peaked in the 55–64 year age group at 2475 pmp for males and 2197 pmp for females. After age 74 years there was a sharp decline in prevalence and very few patients were over 85 years. Most prevalent patients on dialysis were white ethnicity (87%). However, ethnic distribution varied between regions with the highest black to white ratio of 1.9 to 1 in the South.

### Incidence of ESKD in Libya

**Figure 2:**
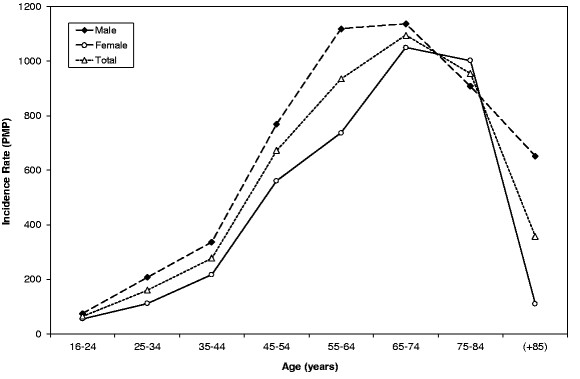
Incidence rate pmp of dialysis-treated ESKD in Libya for males and females by age group.

A total of 1093 new patients started dialysis during the 1-year observation period, giving an incidence rate of 282 pmp. Incidence rates varied between regions with a substantially higher rate observed in the South (Table 1). In most age groups the incidence rate was higher in males than females (Figure 2). The incidence rate increased with age until it peaked in those aged 65–74 years and decreased sharply beyond age 75 years. Incident female patients were slightly older than male patients. Most incident patients on dialysis were white (80.9%).

### Aetiology of ESKD in Libya

Information about primary kidney disease was complete for all prevalent dialysis patients but was missing in 75 (6.9%) of incident dialysis patients. Data are shown in Tables 3 and 4. The most common cause of ESKD among prevalent and incident patients was diabetes. The frequency of different categories of pathology was similar for prevalent and incident patients. Diabetes and hypertensive nephropathy were more common as causes of ESKD in patients aged 50 years or older whereas glomerulonephritis and hereditary diseases were more common in those aged under 50 years. Autoimmune diseases accounted for only a small proportion of all ESKD and were more common among females.

**Table 3:** Aetiology of primary kidney disease of prevalent dialysis patients according to gender and age

**Primary kidney disease**					**Gender**						**Age**	**All patients**
				**Male (n=1402)**	**Female (n=1015)**	**<50 years (n=1268)**	**≥50 years (n=1149)**	**(n=2417)**
Diabetic Nephropathy					396 (28.2)*	245 (24.1)	169 (13.3)*	472 (41.1)	641 (26.5)
Hypertensive Nephropathy					180 (12.8) *	173 (17)	129 (10.2)*	224 (19.5)	353 (14.6)
Glomerulonephritis					318 (22.7) *	194 (19.1)	413 (32.6)*	99 (8.6)	512 (21.2)
Interstitial Nephritis					14 (1)	15 (1.5)	17 (1.3)	12 (1)	29 (1.2)
Obstructive Nephritis					78 (5.6)	42 (4.1)	59 (4.7)	61 (5.3)	120 (5)
Chronic pyelonephritis					19 (1.4) *	29 (2.9)	33 (2.6) *	15 (1.3)	48 (2)
Polycystic kidney disease					82 (5.8)	70 (6.9)	68 (5.4)	84 (7.3)	152 (6.3)
Congenital and hereditary diseases					168 (12) *	130 (12.8)	220 (17.3)*	78 (6.8)	298 (12.3)
Autoimmune diseases					4 (0.3) *	13 (1.3)	16 (1.3) *	1 (0.1)	17 (0.7)
Other					39 (2.8)	32 (3.2)	48 (3.8) *	23 (2)	71 (2.9)
Unknown					104 (7.4)	72 (7.1)	96 (7.6)	80 (7)	176 (7.3)

Data are number (percent).

*P<0.05 versus comparitor group.

**Table 4:** Aetiology of primary kidney disease of incident dialysis patients according to gender and age

**Primary Kidney Disease**	**Gender**		**Age**		**All patients**
	**Male (n=621)**	**Female (n=397)**	**<50 years (n=521)**	**≥50 years (n=497)**	**(n=1018)**
Diabetic Nephropathy	173 (27.9)	116 (29.2)	90 (17.3)*	199 (40)	289 (28.4)
Hypertensive Nephropathy	101 (16.3)	60 (15.1)	29 (5.6)*	132 (26.6)	161 (15.8)
Glomerulonephritis	133 (21.4)	71 (17.9)	173(33.2)*	31 (6.2)	204 (20)
Interstitial Nephritis	7 (1.1)*	17 (4.3)	14 (2.7)	10 (2)	24 (2.4)
Obstructive Nephritis	53 (8.5)*	6 (1.5)	17 (3.3)*	42 (8.5)	59 (5.8)
Chronic pyelonephritis	9 (1.4)	4 (1)	0 (0)*	13 (2.6)	13 (1.3)
Polycystic kidney disease	13 (2.1)*	41 (10.3)	31 (6)	23 (4.6)	54 (5.3)
Congenital and hereditary diseases	42 (6.8)	40 (10.1)	73 (14)*	9 (1.8)	82 (8.1)
Autoimmune diseases	4 (0.6)*	10 (2.5)	11 (2.1)*	3 (0.6)	14 (1.4)
Other	10 (1.6)	4 (1)	10 (1.9)	4 (0.8)	14 (1.4)
Unknown	76 (12.2)*	28 (7.1)	73 (14)*	31 (6.2)	104 (10.2)

Data are number (percent).

*P<0.05 versus comparitor group.

## Discussion

This study represents the first comprehensive description of the epidemiology of dialysis-treated ESKD in Libya. Our study shows a prevalence rate for ESKD markedly higher than rates estimated for the Mediterranean region of 312 to 352 pmp [10,11] despite similar demographic and socioeconomic characteristics between Libya and neighbouring countries. Libya’s prevalence rate is also higher than rates reported from individual countries in the region like Saudi Arabia (475 pmp) [12], El-Minia city in Egypt (208 pmp) [13], Kuwait (240 pmp) [10] and Oman (220 pmp) [10]. Reasons for the high prevalence rate in Libya might include a high prevalence of CKD in the population and limited access to renal transplantation [8,14]. The high incidence rate of ESKD observed in this study (282 pmp) supports the notion that CKD is an important health problem in Libya that requires urgent attention. The rate is more than double that observed in European countries such as Austria (131 pmp) [15] and Denmark (129 pmp) [15] despite the fact that the Libyan population is younger than that of most European countries. Moreover, the incidence rate observed in Libya is even higher than rates reported in other places with young populations such as Qatar (202 pmp) [16], Tunisia (159 pmp) [17], Bhopal city in India (151 pmp) [18], Malaysia (138 pmp) [19], Saudi Arabia (122 pmp) [12], Jordan (111 pmp) [20] and Aleppo city in Syria (60 pmp) [21]. Whereas the prevalence rate did not vary by much in the different geographic regions of Libya the observed incidence rate was substantially higher in the South. Regional variation in incidence of ESKD has been reported by other authors [22,23]. The reasons for this variation in Libya require further investigation and have important implications for future healthcare provision. The South of Libya is a remote, sparsely populated and deprived area. It is also hot and dry and is inhabited by a mixture of different minorities such as black Arabs, Tuareg and Tebou tribes. Thus geographic, racial and socioeconomic factors may all be relevant. It is also notable that the proportion of new patients who commenced dialysis therapy in one year represents 45% of the number of prevalent dialysis patients in Libya. Moreover in the South the incidence rate was triple the prevalence rate. Possible explanations for these observations include a high mortality rate on dialysis or rapidly increasing incidence of ESKD. Both possibilities require urgent investigation and have serious implications for future demand for dialysis in Libya.

The median age of prevalent patients revealed by this study was substantially lower than that observed in other countries including the UK (65.9 years for haemodialysis and 61.2 years for peritoneal dialysis) [24], USA (59.1 years) [25], Iceland (64.4 years), Austria (60.1 years), Denmark (58.8 years) and Greece (58.1 years) [15]. The majority of patients with ESKD in Libya are of economically active age and ESKD therefore has a significant impact on families and society. The observed median age for incident patients in Libya (49 years) was considerably lower than the UK (64.8 years) [26]. Consistent with studies from most other countries, males outnumbered females in the prevalent and incident populations [25,27,28]. Nevertheless, the median age of incident females in the South was a decade younger than females in other regions of the country. This wide range of age presentation in the South might reflect gender inequity, health care deficiency and environmental factors.

Like many other countries diabetic nephropathy was the leading causes of ESKD in both prevalent and incident dialysis populations and was significantly more common among older patients. Generally, the prevalence of diabetes in Middle East region is high according to the International Diabetes Federation [29,30]. Reasons include increasing urbanisation, aging of the population, increasing obesity and falling levels of physical activity [31,32]. Efforts to reduce the burden of ESKD in Libya should therefore focus on measures to reduce incidence of type 2 diabetes mellitus [33,34] through public awareness, screening and promotion of a healthy lifestyle as well as prevention of microvascular complications in those with diabetes [35]. Glomerulonephritis was the second most frequent cause of ESKD in Libya in prevalent and incident cases and was significantly more common among young and male patients. The observed proportion is midway between very high levels reported from countries like China and Kuwait of approximately 35% [36,37], Costa Rica (30%) [38] and Yemen (25%) [39] and lower prevalence observed in countries like Qatar (13%) [16], Sri Lanka (12%) [40] and Pakistan (10%) [41]. Reasons for the high prevalence of glomerulonephritis require further investigation, which is hampered by the shortage of facilities able to perform renal biopsies and histology. A substantial proportion of ESKD was attributed to hypertension but it is unclear what proportion of this represented hypertension secondary to primary renal disease. Congenital and hereditary kidney disease accounted for a significant minority of ESKD in both prevalent and incident patients. This is likely due to the high rate of marriages between relatives, especially first cousins, in Arab communities [42-44] including Libya. A shortage of genetic diagnostics and counselling is a major contributor to this problem. In summary, despite Libya’s unique social and demographic features the observed causes of ESKD are similar to that described in most other countries, though the contribution of glomerulonephritis and hereditary kidney disease is greater than in many developed countries. It is therefore likely that interventions that have proved successful in reducing the incidence of ESKD in other countries such as early detection of CKD, treatment of hypertension with angiotensin-converting enzyme inhibitors and prevention or treatment of diabetes are likely to be effective in Libya too.

Strengths of this study are the inclusion of all current dialysis units in the country to ensure collection of the most complete data possible and prospective capture of data regarding new patients commencing dialysis. Limitations include reliance on limited and partially incomplete medical records and lack of histology to confirm the cause of ESKD in the majority of cases. We have described the epidemiology of ESKD among patients receiving dialysis but there are no data regarding patients with ESKD who died without the benefit of dialysis. It is therefore likely that the true incidence of ESKD is higher than what we have observed. Further research is required to investigate how many patients with ESKD are not offered dialysis and the reasons for this.

## Conclusions

In conclusion we report for the first time high prevalence and incidence rates of dialysis-treated ESKD in Libya. The relatively young age of those affected emphasizes the need for urgent measures to reduce the incidence of CKD and its progression. Emphasis should be placed in the first instance on the prevention and treatment of diabetes and hypertension. Further research is required to investigate the high incidence of glomerulonephritis. As Libya rebuilds its healthcare system following the recent conflict it is hoped that the prevention of ESKD will constitute an important priority and that the data presented will inform the strategies required.

## Methods

The study was performed by a Libyan researcher with support and guidance provided by a Nephrology Department in the United Kingdom. First, a cross-sectional study was performed from May to September 2009. This included all adult maintenance dialysis facilities in Libya (n=39), which all belong to the public sector. Medical supervisors of maintenance dialysis facilities throughout the country were contacted by visit or phone call to be informed about the study. All agreed to participate and to cooperate with the research team.

A data collection sheet was prepared and used to gather information about all currently registered dialysis patients in the country. Forms included data regarding date of birth, sex, ethnicity, nationality, type of dialysis, date of dialysis initiation and primary kidney disease. Date of birth was recorded if known. Otherwise, the year of birth was used. The same applied for the date of first dialysis. Primary kidney disease was recorded according to the opinion of the treating physician or medical reports when available.

For the majority of dialysis units (n=28), the research team collected all information by interviewing patients and inspecting medical records to assure quality and validity of the data. For remote facilities, data collection sheets were distributed to the clinical supervisor for completion. Frequent phone calls were used to answer queries and ensure that data were correctly documented. A pilot study was undertaken in 5 dialysis units that included 100 patients (20 from each unit). Difficulties in collecting data were explored and solutions developed. Data were cross checked for patients who were known to receiving dialysis in another centre within the country. Patients who were absent at the time of the study for a period of less than one month for travel in or outside the country were included among the prevalent population. Analyses performed included all adult patients prevalent on dialysis on 30 August 2009.

Population estimates were obtained from the Libyan national statistics department. The age and gender breakdown of the population in each region was obtained from mid-2009 population estimates based on 2006 census data. The number of dialysis patients was calculated for the country as a whole. In order to investigate geographic variation in the epidemiology of ESKD, data were also grouped into 3 main geographic regions.

The second stage of the study was a prospective collection of information about all new patients who started dialysis from September 2009 to August 2010. Variables collected were identical to that used during the cross-sectional study and the same data forms were used. The research team conducted data collection frequently during the year to ensure the inclusion of every new patient in the country. Subsequently, researchers conducted field visits to dialysis facilities from September to December 2010 to validate the information.

Data are presented as mean±standard deviation if normally distributed or median (interquartile range) if not. A Chi-squared test was used to compare frequencies between groups. A p-value of <0.05 was considered statistically significant. Analysis was performed by using SPSS version 16.0.

Permission to conduct the study was granted by the Ministry of Health. Ethical approval for the research was obtained from the Libyan National Committee for Bioethics and Bio-safety. Patients each gave written informed consent.

## Competing interest

The authors have no conflicts of interest to declare. The results presented in this paper have not been published previously in whole or part, except in abstract form.

## Authors’ contributions

The contribution of each of the authors was as follows: WA: study design, collection of all data, analysis of data, writing of manuscript. CWM: study design, review of data, writing and revision of manuscript. MWT: study design, review of data, writing and revision of manuscript. All authors read and approved the final manuscript.

## Authors’ information

WA is based in Libya and conducted this study as part of a PhD project, supervised by CWM and MWT, who are consultant nephrologists in the UK.

## Pre-publication history

The pre-publication history for this paper can be accessed here:

http://www.biomedcentral.com/1471-2369/13/33/prepub
